# Supplementary material for: Functionalized Crystalline N-Trimethyltriindoles: Counterintuitive Influence of Peripheral Substituents on Their Semiconducting Properties
Source: Molecules. 2022 Feb 8;27(3):1121. doi: 10.3390/molecules27031121 (PMC8839582; doi:10.3390/molecules27031121)
Supplement: Supplementary file 1 [file molecules-27-01121-s001.zip › molecules-1551467-supplementary.pdf]

Supporting Information

Functionalized Crystalline *N*-trimethyltriindoles: Counterintuitive Influence of Substituents on their Semiconducting Properties

Sergio Gámez-Valenzuela [1], Angela Benito-Hernández[2], Marcelo Echeverri[2], Enrique Gutierrez-Puebla[2], Rocío Ponce Ortiz [1], M. Carmen Ruiz Delgado [1,*] and Berta Gómez-Lor [2,*]

[1] Department of Physical Chemistry, University of Málaga, Campus de Teatinos s/n, 29071, Málaga, Spain.; carmenrd@uma.es

[2] Instituto de Ciencia de Materiales de Madrid-Consejo Superior de Investigaciones Científicas (ICMM-CSIC), Sor Juana Inés de la Cruz 3, Cantoblanco 28049, Madrid, Spain.; bgl@icmm.csic.es

Table of Contents

	Page
**1. Copy of ^{1}HNMR and ^{13}CNMR spectra of 2 and 3**	S2
**2. Single crystal X-ray and refinement data of 2**	S4
**3. Powder X-ray diffractogram of 3**	S4
**4. DFT calculations**	S5
**5. OFET derived electrical data**	S11
**6. Morphologic characterization**	S13

1. Copy of ^{1}HNMR and ^{13}CNMR spectra of 2 and 3

^{1}H NMR (300MHz, $CDCl_3$, 25ºC)

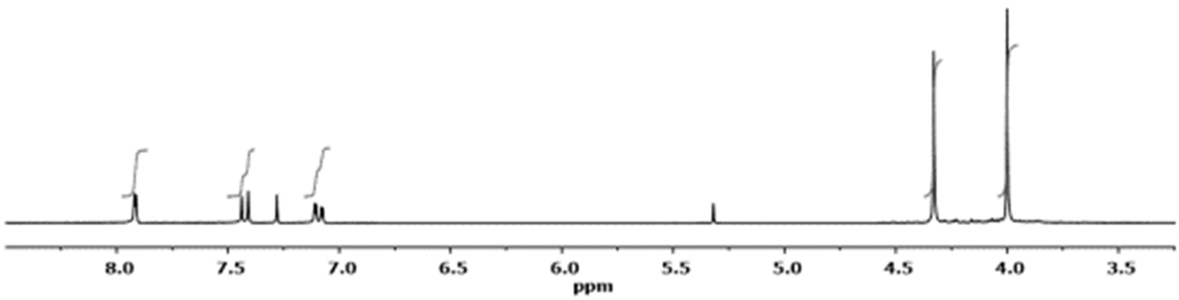

^{13}C NMR (300MHz, $CDCl_3$, 25ºC)

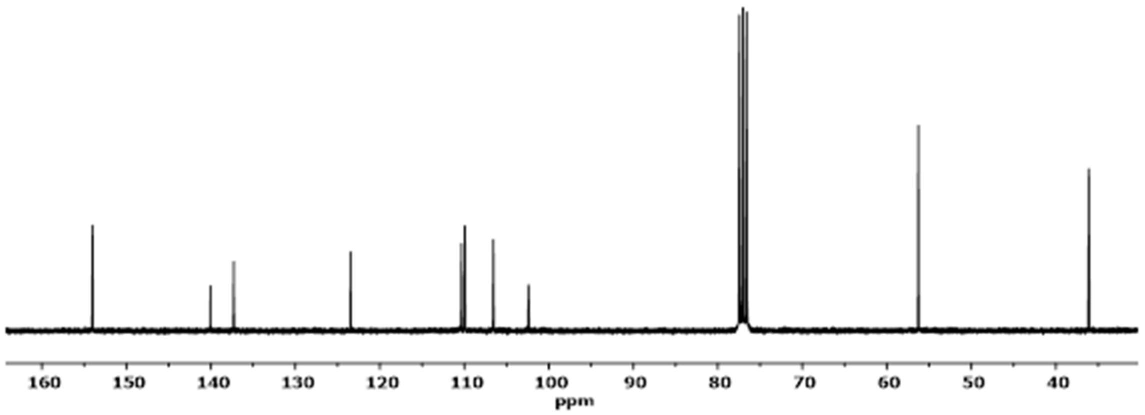

^{1}HNMR spectrum of 3,8,13-triacetyl-5,10,15-trimethyl-10,15-dihydro-5*H*-diindolo[3,2-*a*:3',2'-*c*]carbazole (3).

^{1}H NMR (300MHz, $C_2D_2Cl_4$, 100ºC)

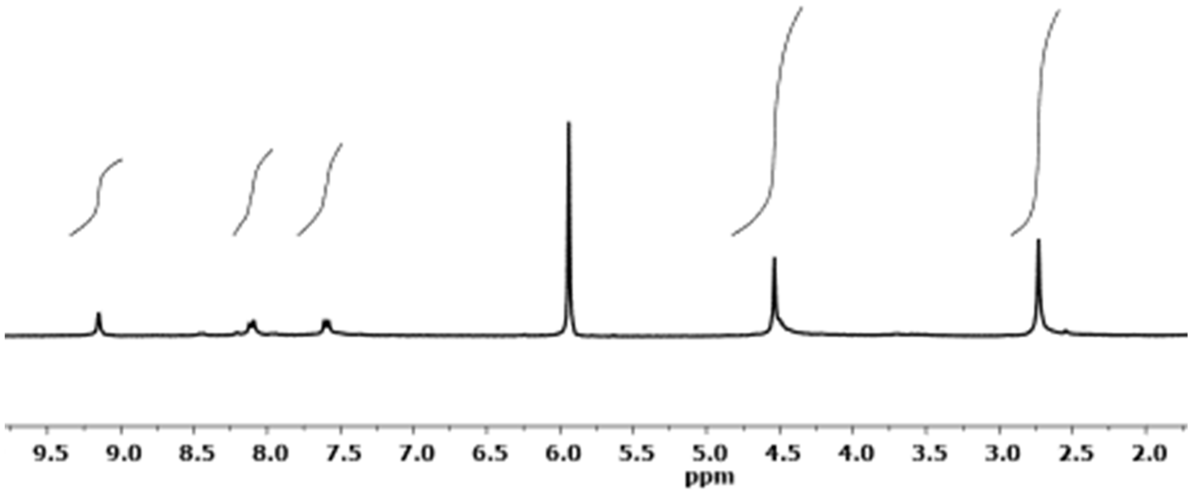

^{13}C NMR (75MHz, $C_2D_2Cl_4$, 100ºC)

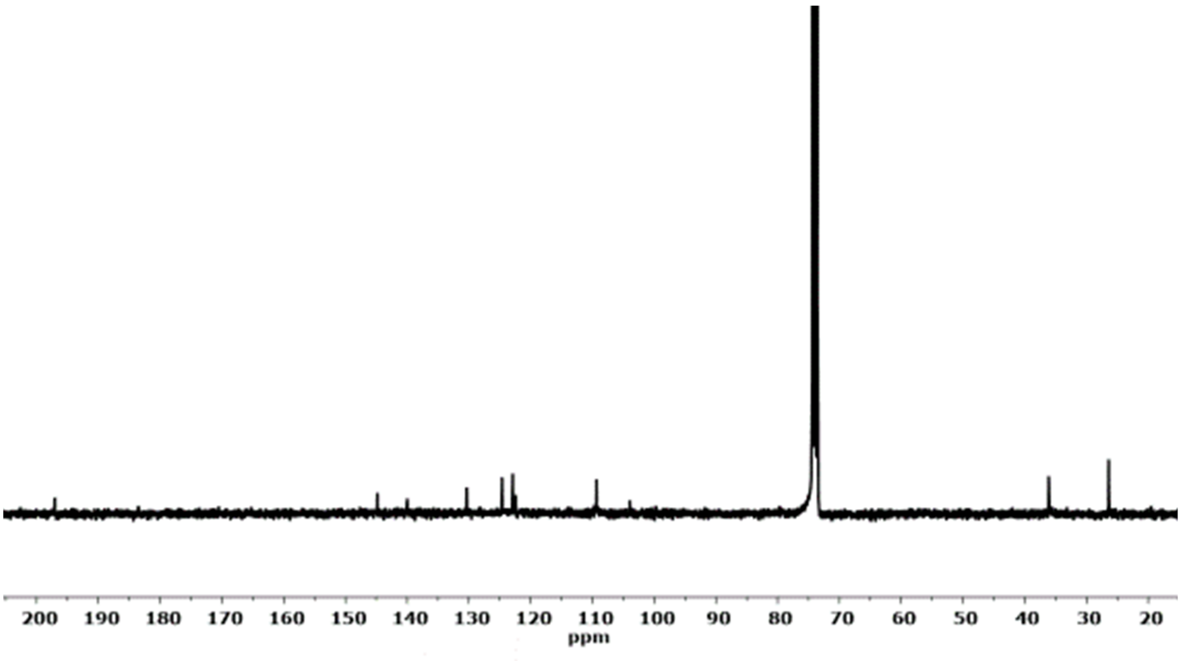

2. Single crystal X-ray and refinement data of 2

Table S1. Main crystallographic and refinement data for **2**.

Compound	**2**
Formula	C30 H27 N3 O3
Molecular Weight /gmol^{-1}	477.55
Temperature/K	223(2)
Wavelength/Å	1.54178
Crystal System	Monoclinic
Space Group	*P63*
a/Å	13.8394(9)
b/Å	13.8394(9)
c/Å	6.9759(8)
α/°	90
β/°	90
γ/°	120
Z	2
Dx/ g.cm^{-3}	1.371
µ/mm-1	0.717
Final R indexes [I>2σ(I)]	R1: 0.1036<br>wR2: 0.3071

3. Powder X-ray diffractogram of 3

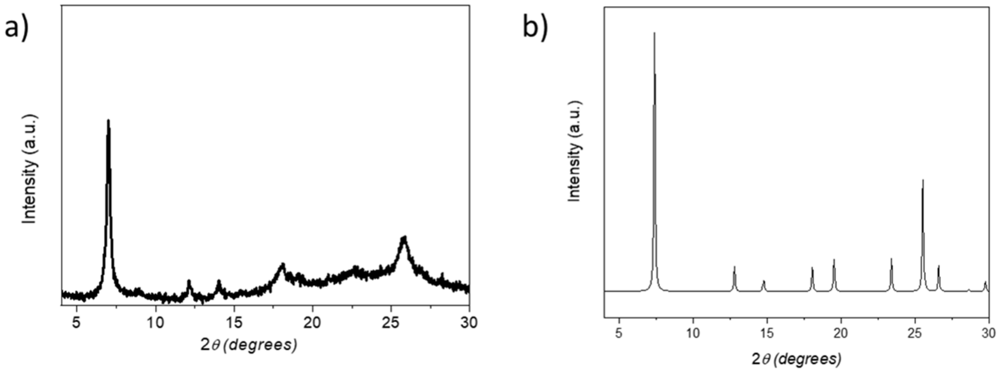

Figure S1. a) Experimental powder X-ray diffractogram of **3** and b) simulated X-ray diffractogram of **2** obtained from its single crystal data.

4. DFT Calculations

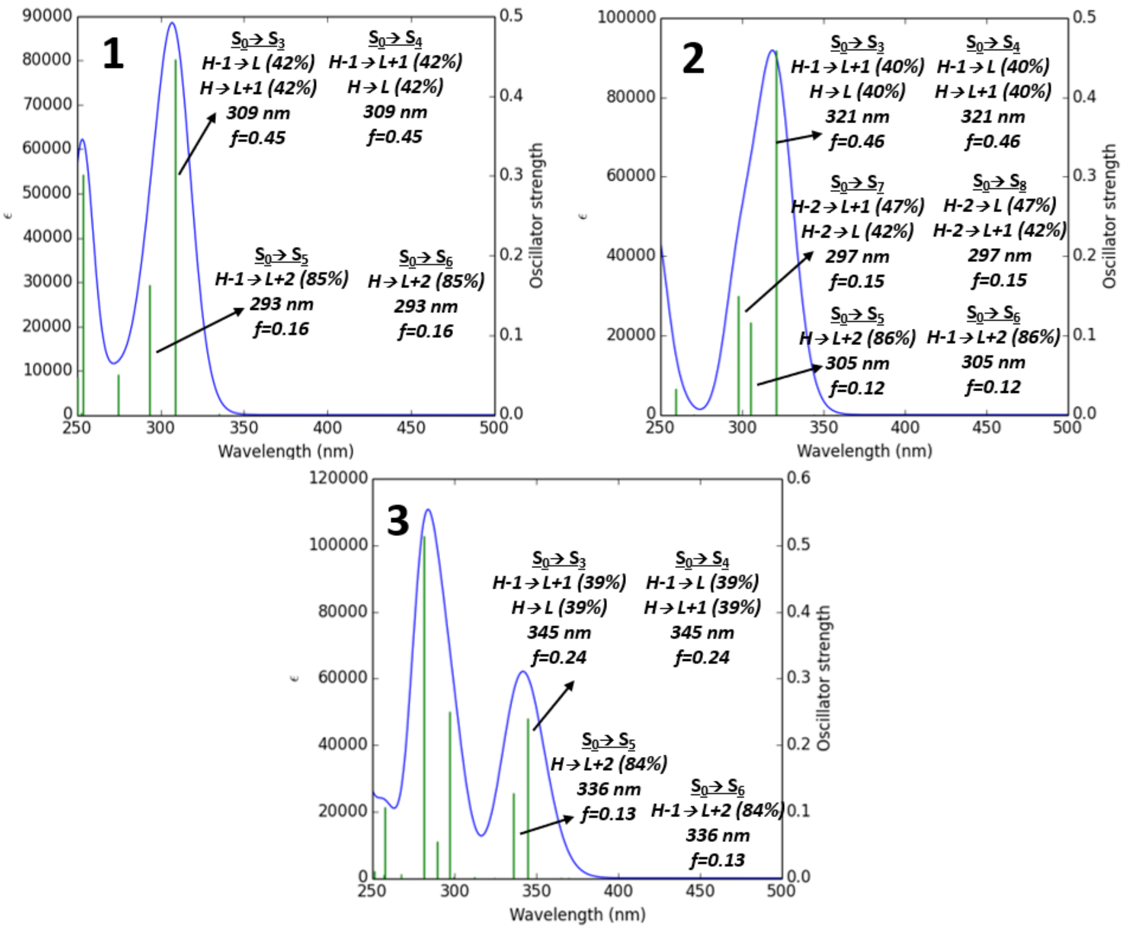

Figure S2. Simulated absorption spectra and main excitations (shown as vertical bars) for the compounds under study. The computations were done at the TD-DFT level by using B3LYP functional and 6-31G** basis set.

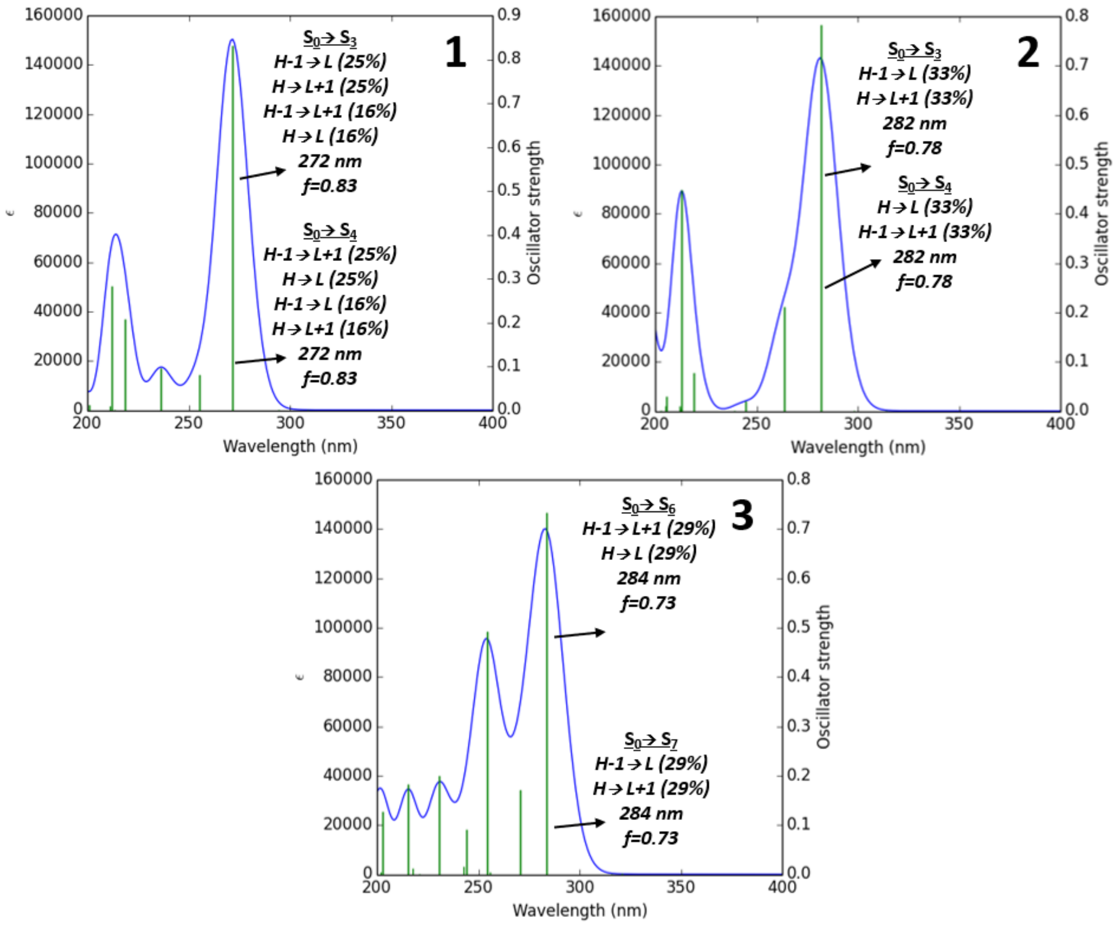

Figure S3. Simulated absorption spectra and main excitations (shown as vertical bars) for the compounds under study. The computations were done at the TD-DFT level by using M06-2X functional and 6-31G** basis set.

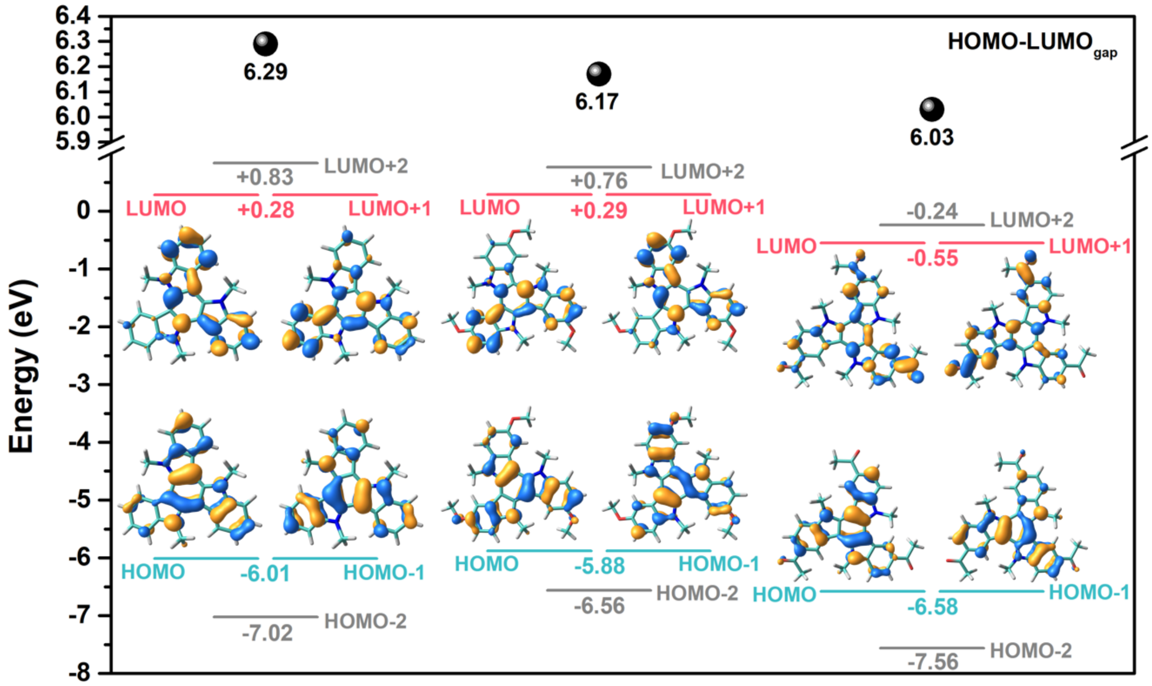

Figure S4. DFT-calculated FMOs energies and topologies for all the compounds under study at the M06-2X/6-31G** level of theory.

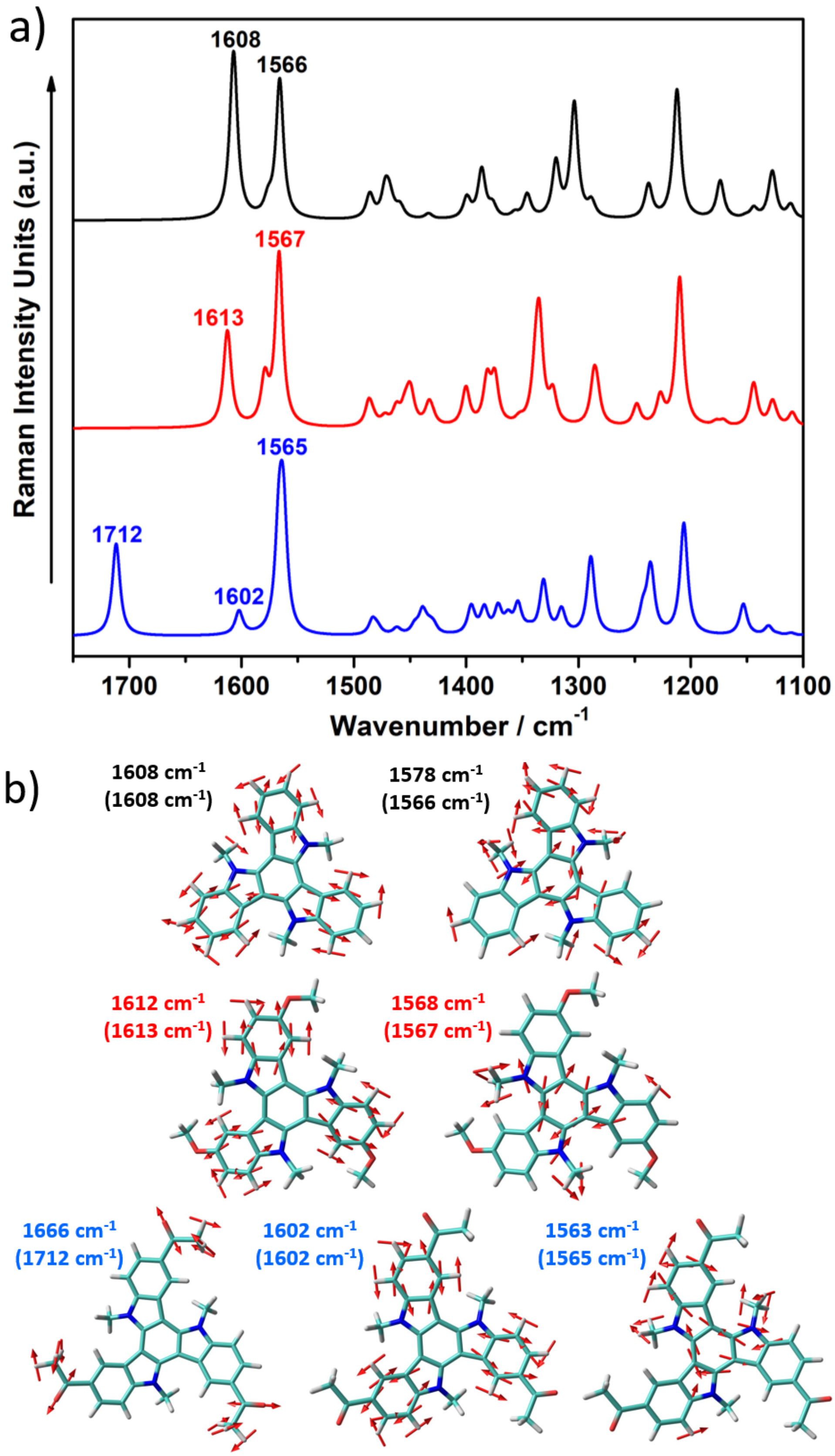

Figure S5. Theoretical (a) Raman spectrum and (b) vibrational eigenvectors associated with the most outstanding C=C/C-C Raman features for the previously optimized structures of **1** (top), **2** (middle) and **3** (bottom) compounds at B3LYP/6-31G** level. The experimental and theoretical (in parentheses) wavenumbers are also shown.

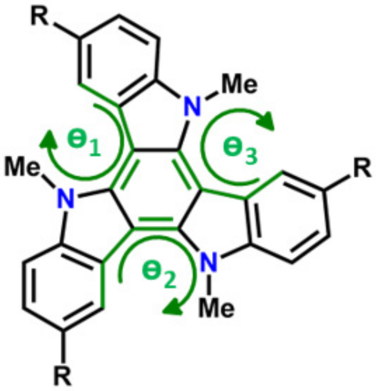

Compound	Neutral State		
	θ_1	θ_2	θ_3
**1**	8º (8º)	8º (8º)	8º (8º)
**2**	8º (8º)	8º (8º)	8º (8º)
**3**	9º (8º)	7º (8º)	8º (8º)

Figure S6. DFT-calculated (B3LYP/6-31G**) dihedral angles values along the conjugated backbone for the triindole systems under study. Values in parenthesis correspond to those calculated at the M06-2X/6-31G** level.

a)

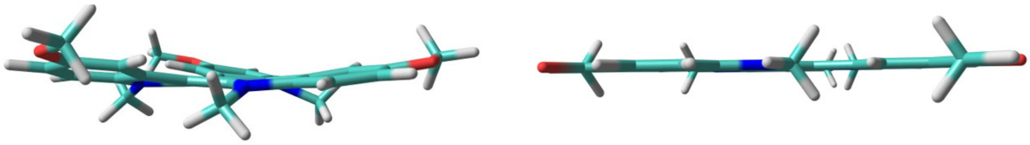

Relaxed (twisted) Constrained (planar)

b)

	B3LYP/6-31G**			M06-2X/6-31G**		
	$E_{TWISTED}$ (HF)	E_{PLANAR} (HF)	ΔE (Kcal/mol)	$E_{TWISTED}$ (HF)	E_{PLANAR} (HF)	ΔE (Kcal/mol)
**1**	-1205.85420369	-1205.84415131	6.3	-1205.36423168	-1205.35344835	6.8
**2**	-1549.42151750	-1549.41095946	6.6	-1548.79765866	-1548.78635434	7.1
**3**	-1663.80909824	-1663.79954533	6.0	-1663.13519518	-1663.12502080	6.4

Figure S7. a) Lateral view of DFT-computed global minimum structure for the fully relaxed (twisted) and constrained (planar) geometries of triindole **1** taken as an example. b) Energy differences (ΔE) between the fully relaxed (twisted) and constrained (planar) geometries of triindoles **1**-**3**.

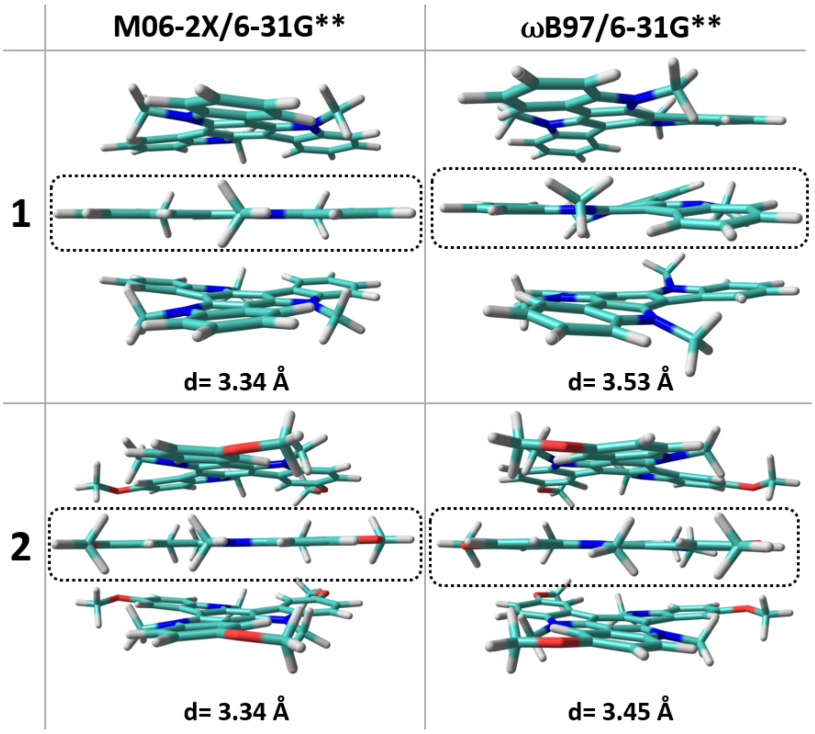

Figure S8. DFT-computed global minimum structures for a trimer model of **1** and **2** at the M06-2X and ωB97X level. The average centroid-centroid distance of the central aromatic rings between adjacent molecules is also shown.

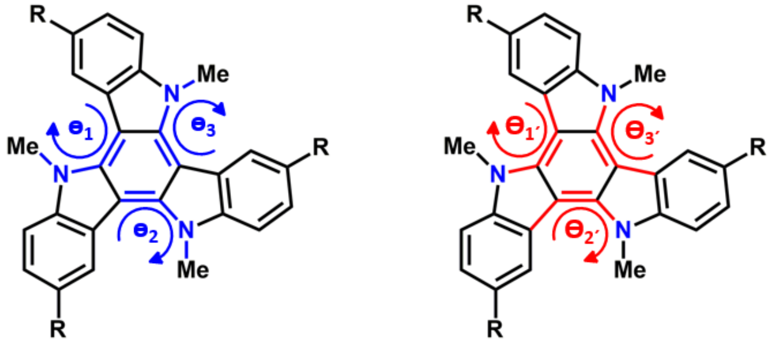

	Neutral State			Radical Cation			Δθ (Neutral-Cation states)		
	θ_1	θ_2	θ_3	θ_1	θ_2	θ_3	$\Delta\theta_1$	$\Delta\theta_1$	$\Delta\theta_1$
1	25º	25º	25º	18º	26º	17º	7º	-1º	7º
2	27º	27º	27º	19º	27º	19º	8º	0º	8º
3	24º	24º	21º	20º	23º	16º	4º	1º	5º

	Neutral State			Radical Cation			Δθ (Neutral-Cation states)		
	$\theta_1´$	$\theta_2´$	$\theta_3´$	$\theta_1´$	$\theta_2´$	$\theta_3´$	$\Delta\theta_1$	$\Delta\theta_1$	$\Delta\theta_1$
1	6º	6º	6º	6º	12º	3º	0º	-6º	3º
2	6º	6º	6º	7º	11º	3º	-1º	-5º	3º
3	6º	7º	6º	8º	10º	2º	-2º	-3º	4º

Figure S9. DFT-calculated dihedral angles values along the conjugated backbone for the triindole systems under study on their neutral and radical cation states at the B3LYP/6-31G** level.

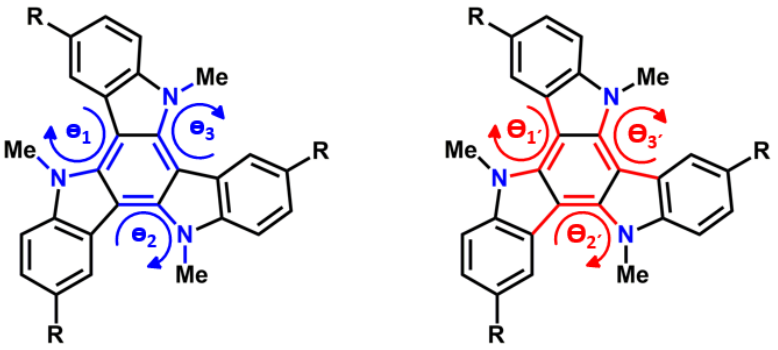

	Neutral State			Radical Cation			Δθ (Neutral-Cation states)		
	θ_1	θ_2	θ_3	θ_1	θ_2	θ_3	$\Delta\theta_1$	$\Delta\theta_1$	$\Delta\theta_1$
1	25°	25°	25°	15°	25°	19°	10°	0°	6°
2	26°	26°	26°	17°	26°	18°	9°	0°	8°
3	24°	24°	24°	15°	25°	19°	9°	-1°	5°

	Neutral State			Radical Cation			Δθ (Neutral-Cation states)		
	θ_1´	θ_2´	θ_3´	θ_1´	θ_2´	θ_3´	$\Delta\theta_1$	$\Delta\theta_1$	$\Delta\theta_1$
1	6°	6°	6°	7°	1°	11°	-1°	5°	-5°
2	6°	6°	6°	7°	1°	12°	-1°	5°	-6°
3	6°	6°	6°	7°	1°	10°	-1°	5°	-4°

Figure S10. DFT-calculated dihedral angles values along the conjugated backbone for the triindole systems under study on their neutral and radical cation states at the M06-2X/6-31G** level.

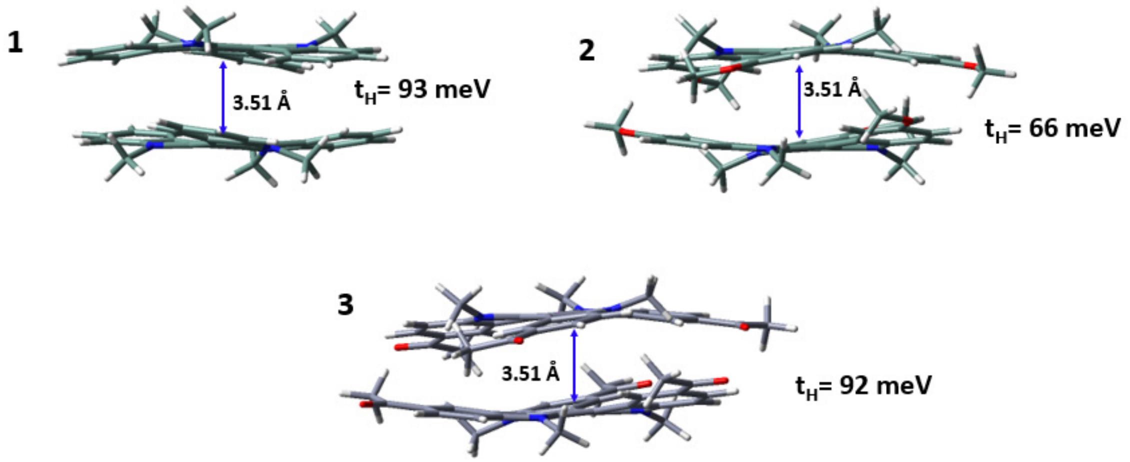

Figure S11. DFT-estimates of the transfer integrals (in meV) for holes for model cofacial dimers of **1**-**3** systems.

5. OFETs derived electrical data

Table S2. OFET derived electrical data for **1** vapor-deposited films under different conditions.

Subst. treatment	Substrate temp. (^oC)		μ_h ($cm^2 V^{-1} s^{-1}$)	I_{ON}/I_{OFF}	V_T(V)
OTS	60	Average	2.2×10^{-2}	1×10^7	-11
		**Best**	**2.8×10^{-2}**	**3×10^7**	**-17**
	25	Average	2.0×10^{-5}	5×10^3	-3
		**Best**	**3.5×10^{-5}**	**2×10^4**	**0**
HMDS	60	Average	3.2×10^{-4}	1×10^5	-11
		**Best**	**3.6×10^{-4}**	**2×10^5**	**-6**
	25	Average	1.2×10^{-4}	3×10^4	-14
		**Best**	**1.3×10^{-4}**	**9×10^4**	**-8**
No treatment	60	Average	1.9×10^{-5}	3×10^3	8
		**Best**	**2.8×10^{-5}**	**1×10^4**	**6**
	25	Average	5.9×10^{-6}	2×10^2	-2
		**Best**	**6.5×10^{-6}**	**3×10^2**	**0**

Table S3. OFET derived electrical data for **2** vapor-deposited films under different conditions.

Subst. treatment	Substrate temp. (^oC)		μ_h ($cm^2 V^{-1} s^{-1}$)	I_{ON}/I_{OFF}	V_T(V)
OTS	60	Average	2.2×10^{-5}	2×10^2	3
		**Best**	**5.1×10^{-5}**	**3×10^2**	**3**
	25	Average	1.9×10^{-4}	9×10^5	-5
		**Best**	**2.0×10^{-4}**	**2×10^5**	**-5**
	80	Average	9.3×10^{-4}	3×10^3	13.1
		**Best**	**1.3×10^{-3}**	**6×10^3**	**-2**
	90	Average	1.4×10^{-3}	6×10^3	-4
		**Best**	**1.6×10^{-3}**	**1×10^4**	**-8**
HMDS	60	Average	5.8×10^{-6}	6×10^1	-7
		**Best**	**6.6×10^{-6}**	**2×10^2**	**-5**
	25	Average	8.2×10^{-6}	1×10^2	-10
		**Best**	**9.8×10^{-6}**	**3×10^2**	**-7**
	80	Average	7.5×10^{-6}	1×10^3	4
		**Best**	**8.3×10^{-6}**	**6×10^3**	**-8**
No treatment	60	Average	1.9×10^{-5}	3×10^3	8
		**Best**	**2.8×10^{-5}**	**1×10^4**	**6**
	25	Average	5.9×10^{-6}	2×10^2	-2
		**Best**	**6.5** $\times 10^{-6}$	**3×10^2**	**0**
	80	Average	1.8×10^{-6}	9	20
		**Best**	**2.1×10^{-6}**	**1×10^1**	**10**

Table S4. OFET derived electrical data for **3** vapor-deposited films under different conditions.

Subst. treatment	Substrate temp. (°C)		μ_h ($cm^2 V^{-1} s^{-1}$)	I_{ON}/I_{OFF}	V_T(V)
**OTS**	**25**	Average	1.0×10^{-5}	8×10^{1}	-14
		**Best**	**2.2×10^{-5}**	**2×10^{2}**	**-29**
	**65**	Average	1.3×10^{-5}	9×10^{1}	-12
		**Best**	**1.7×10^{-5}**	**2×10^{2}**	**-29**
	**120 (post annealing)**	Average	3.1×10^{-5}	2×10^{2}	-17
		**Best**	**4.5×10^{-5}**	**3×10^{2}**	**-21**
**HMDS**	**25**	Average	1.7×10^{-5}	4×10^{2}	-18
		**Best**	**2.2×10^{-5}**	**1×10^{3}**	**-26**
	**65**	Average	1.5×10^{-5}	2×10^{2}	-19
		**Best**	**1.7×10^{-5}**	**2×10^{2}**	**-12**
	**120 (post annealing)**	Average	1.8×10^{-5}	1×10^{2}	-13
		**Best**	**2.2×10^{-5}**	**2×10^{2}**	**-23**
**No. treatment**	**25**	Average	6.4×10^{-6}	6×10^{1}	-8
		**Best**	**9.9×10^{-6}**	**1×10^{2}**	**-11**
	**65**	Average	6.5×10^{-6}	4×10^{1}	-3
		**Best**	**7.8×10^{-6}**	**7×10^{1}**	**-5**
	**120 (post annealing)**	Average	1.0×10^{-6}	1×10^{1}	18
		**Best**	**7.8×10^{-6}**	**2×10^{1}**	**7**

6. Morphologic characterization

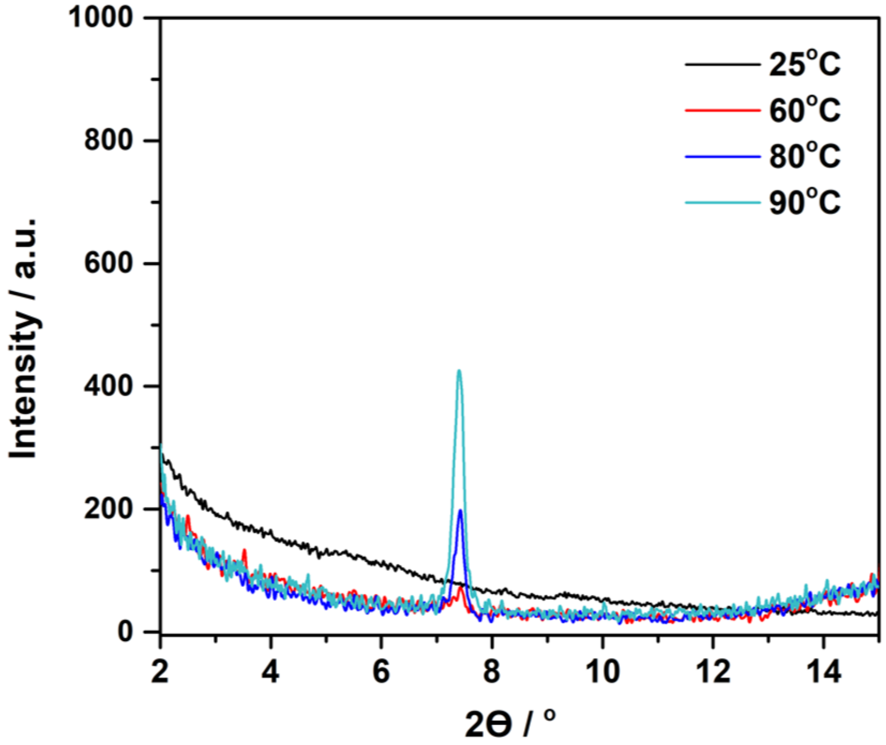

Figure S12. Experimental GIXRD spectra of triindole **2** deposited by slow sublimation under vacuum conditions on OTS treated Si/SiO_2 substrates preheated at different temperatures.

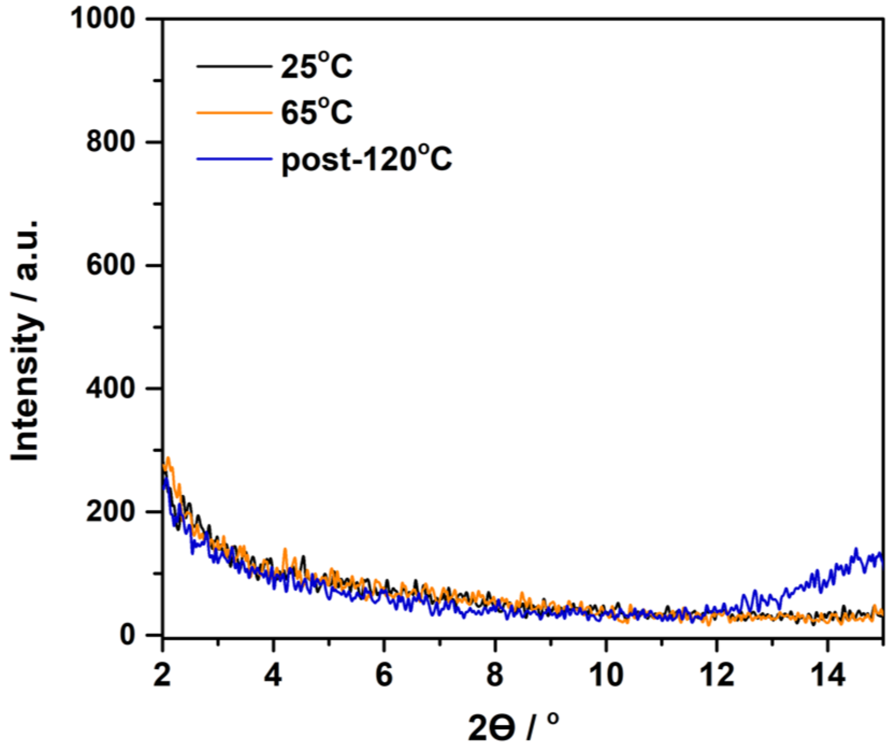

Figure S13. Experimental GIXRD spectra of triindole **3** deposited by slow sublimation under vacuum conditions on OTS treated Si/SiO_2 substrates preheated at different temperatures and with a post-annealing of 2 hours at 120°C (blue).

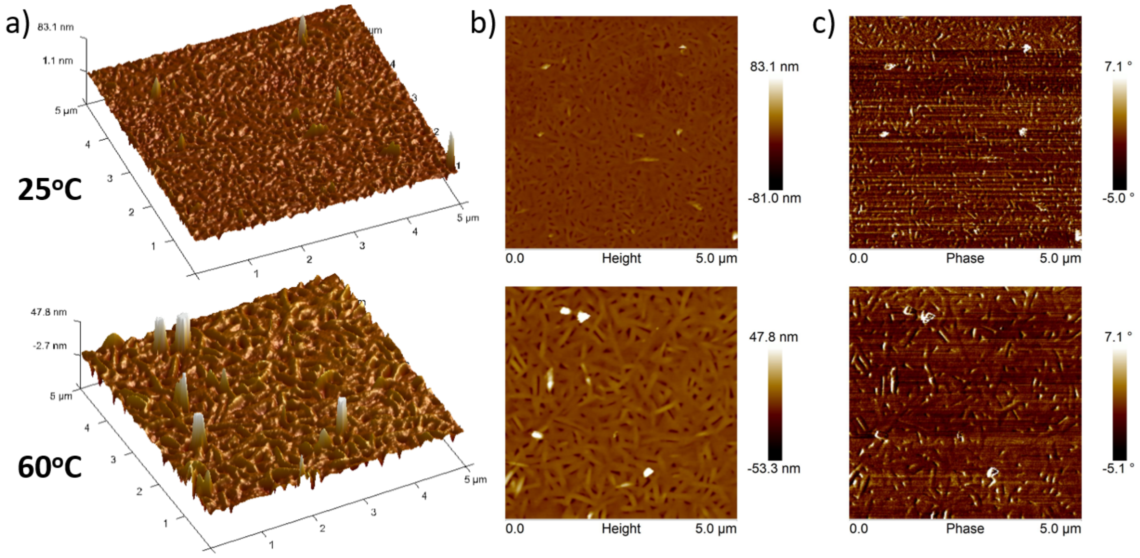

Figure S14. Tapping-mode AFM images of (a) 3D-topography, (b) height and (c) phase in 5 µm x 5 µm scan size of thin films of triindole **1** deposited on OTS-treated Si/SiO_2 substrates at different temperatures.

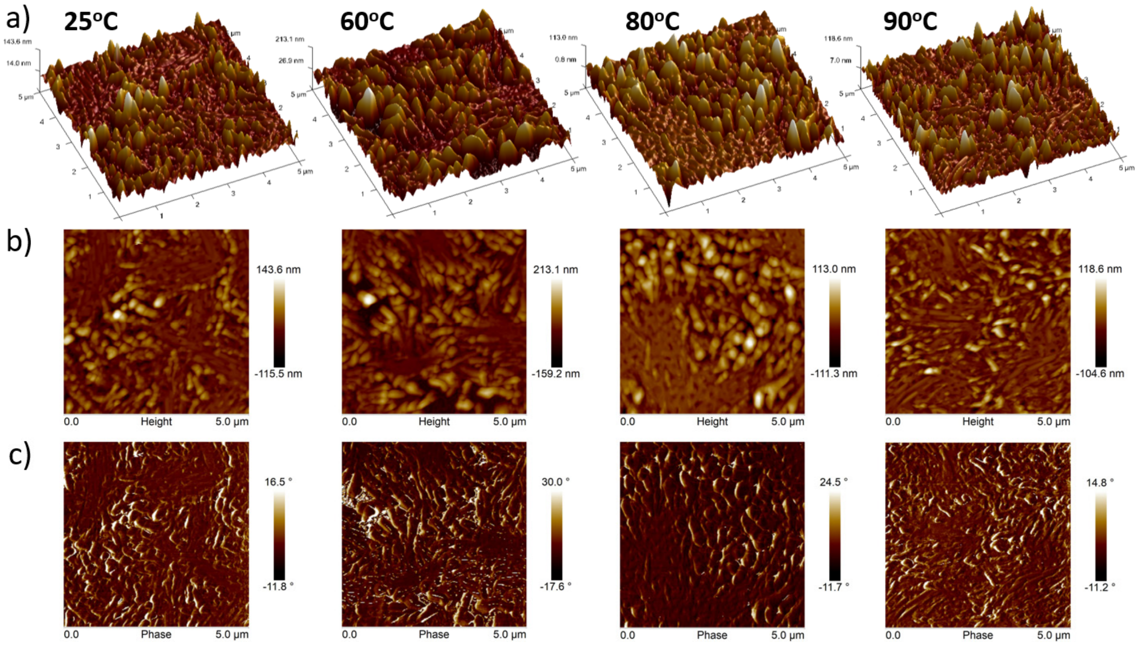

Figure S15. Tapping-mode AFM images of (a) 3D-topography, (b) height and (c) phase in 5 µm x 5 µm scan size of thin films of triindole **2** deposited on OTS-treated Si/SiO2 substrates at different temperatures.

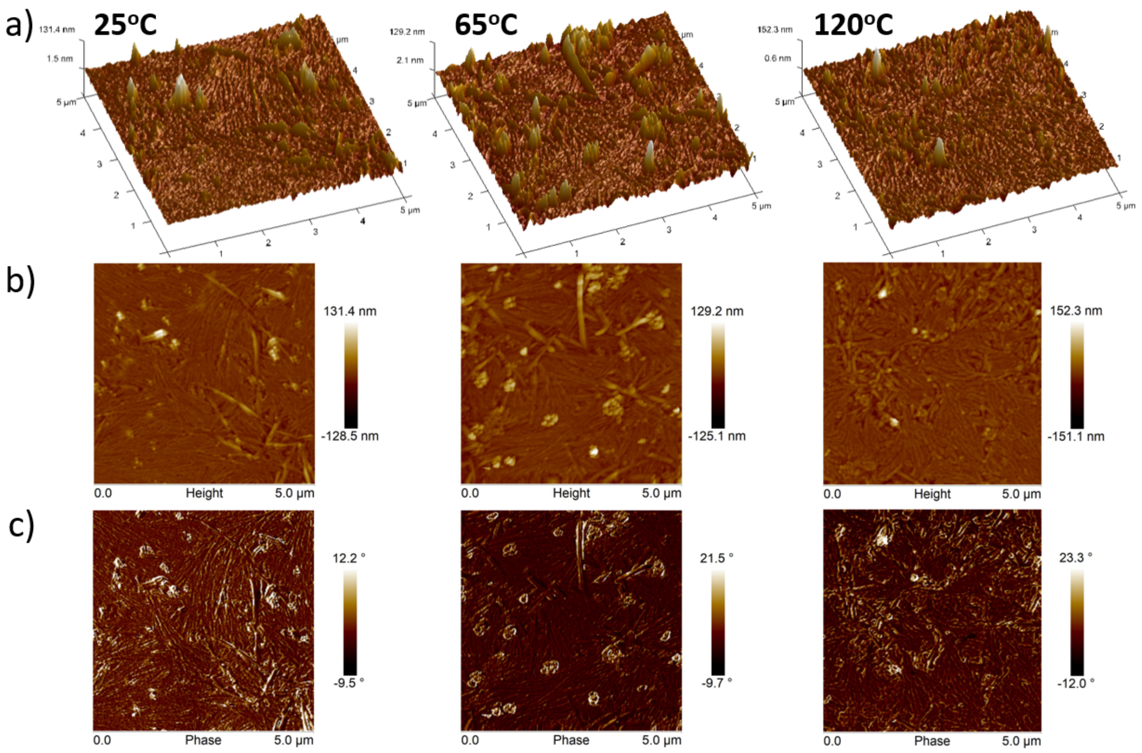

Figure S16. Tapping-mode AFM images of (a) 3D-topography, (b) height and (c) phase in 5 µm x 5 µm scan size of thin films of triindole **3** deposited on OTS-treated Si/SiO2 substrates at different temperatures.
